# Simple and Robust Microfabrication of Polymeric Piezoelectric Resonating MEMS Mass Sensors

**DOI:** 10.3390/s22082994

**Published:** 2022-04-13

**Authors:** Chang Ge, Edmond Cretu

**Affiliations:** Department of Electrical and Computer Engineering, Faculty of Applied Science, The University of British Columbia, Vancouver, BC V6T 1Z4, Canada; edmondc@ece.ubc.ca

**Keywords:** polymeric thin-film MEMS, PVDF-TrFE, piezoelectric MEMS, resonating MEMS, mass sensor, laser micromachining, adhesive lamination

## Abstract

Resonating MEMS mass sensors are microdevices with broad applications in fields such as bioscience and biochemistry. Their advantageous surface-to-volume ratio makes their resonant frequency highly sensitive to variations in their mass induced by surface depositions. Recent global challenges, such as water quality monitoring or pandemic containment, have increased the need for low-cost (even disposable), rapidly fabricated microdevices as suitable detectors. Resonant MEMS mass sensors are among the best candidates. This paper introduces a simple and robust fabrication of polymeric piezoelectric resonating MEMS mass sensors. The microfabrication technology replaces the traditional layer-by-layer micromachining techniques with laser micromachining to gain extra simplicity. Membrane-based resonant sensors have been fabricated to test the technology. Their characterization results have proven that the technology is robust with good reproducibility (around 2% batch level variations in the resonant frequency). Initial tests for the MEMS mass sensors’ sensitivity have indicated a sensitivity of 340 Hz/ng. The concept could be a starting point for developing low-cost MEMS sensing solutions for pandemic control, health examination, and pollution monitoring.

## 1. Introduction

Microelectromechanical systems (MEMS) are microstructures able to couple the mechanical and electrical energy domains at microscales. Their micrometer-scale dimensions favor an amplified coupling efficiency which gives MEMS devices high performance for many applications. One representative example in this respect is resonating MEMS mass sensors.

Resonating MEMS mass sensors generally have two main advantages. First, they have high sensitivity. MEMS resonators usually have nanogram-level (ng, 10^−9^ g) original mass and resonant frequencies ranging from 10 kHz to 100 MHz. A significant resonant frequency shift of MEMS resonators can be triggered by a mass variation at the nanogram (10^−9^ g), picogram (10^−12^ g), or even femtogram (10^−15^ g) level [1]. Secondly, their resonant behavior usually has a narrow bandwidth, providing robustness against noise sources. Mechanical-thermal noises are common in MEMS devices [2]. They are distributed over the whole frequency band. There are usually two dominant noise components limiting the sensing resolution: a wide thermomechanical white noise and a 1/f pink noise component, dominant at low frequencies. When a MEMS device operates in a wide frequency band, the spectral noise distribution is integrated over the entire bandwidth, limiting the achievable resolution. However, a resonating MEMS mass sensor operates in a narrow spectral bandwidth centered around its resonant frequency. The impact of the 1/f strong noise components and the sensors’ corresponding total equivalent noise output is reduced. The advantages of resonating MEMS mass sensors have enabled a wide range of applications. They can detect material at different quantity levels. The target quantity level leads to different design strategies and, correspondingly, different performances, as briefly summarized in Table 1.

Table 1 classifies resonating MEMS mass sensors into two complementary types. Suspended microfluidic resonators (SMR) are MEMS resonators with internal microfluidic channels. They load the liquid sample into the device’s body. In such a way, SMRs can maintain a high-quality factor when analyzing liquids. Suspended microfluidic channels are usually fabricated using the micromachining processes of silicic materials. Most SMRs are packaged in a vacuum. As a result, SMRs usually have ultra-high sensitivities. Existing research on SMRs has reported sensitivity at the level of picogram (10–12 g), femtogram (10–15 g), or even yoctogram (10–24 g) [1,8,9,10]. Due to their ultra-high sensitivity, SMRs are powerful tools to characterize a single or a few particles in chemical and biomedical fields. However, such high performance comes with delicate yet complex microfabrication flows and correspondingly high costs.

MEMS resonators without microfluidic channels can also be used as mass sensors. These MEMS mass sensors usually work in a two-step manner. First, they are exposed to an ambient, either gaseous or liquid, to capture the target material on their surface. This process is usually implemented with a functional coating layer [3,4,11,12,13]. Then, the measurement of the mechanical resonance for sensing purposes is conducted. The complexity of microfabrication flows for the two-step MEMS mass sensors is lower than the microfabrication flows for SMRs, thanks to their simpler structure designs. The selection of structural materials for these two-step mass sensors is also diverse. In addition to silicon, polymers have been used to make these two-step MEMS mass sensors [4]. Furthermore, these two-step MEMS resonating mass sensors are usually not packaged in a vacuum. As a trade-off, their sensitivity is lower than SMRs, usually at the nanogram (*ng*) level. They are usually used to measure particle concentration [3,4,14,15,16,17]. This application is becoming increasingly important in recent years.

Accurate particle concentration measurement is essential to addressing global challenges such as pandemic containment and the monitoring of water toxicity and pollution levels. As a recent trend, sensors dedicated to tackling these challenges have to fulfill the demand of being easily accessible or disposable. One way to fulfill these demands is to develop low-cost, rapid, and robust microfabrication flows for concentration sensors. In this respect, polymeric two-step MEMS mass sensors have high potential due to their unique advantages related to microfabrication technologies. Polymer materials have high flexibility in the selection of processing technologies. Some direct micromachining techniques can form polymeric microstructures with a higher speed and lower cost.

This paper reports a new research effort related to the rapid, low-cost, and robust fabrication of polymeric resonating MEMS mass sensors. The paper focuses on two aspects: (1) The development and validation of rapid polymeric MEMS fabrication technologies based on direct micromachining techniques, and (2) The validation of the performance of the fabricated MEMS resonating mass sensors using methods similar to existing research works on similar topics. The microfabrication flow developed in this paper combines polymer laser micromachining with 3D printing and adhesive lamination to replace the traditional micromachining flows. The purpose is to reduce the process complexity and the corresponding cost. The robustness of the new fabrication flow is experimentally validated. Then, it is used to fabricate polymeric MEMS mass sensors. A function test has been conducted to evaluate the performance of the sensors. The corresponding results demonstrate these facts: (1) The new microfabrication flow is reliable, represented by good reproducibility. (2) The polymeric piezoelectric resonating MEMS mass sensors have acceptable performance, even without an in-depth optimization of the structural designs.

For the rest of the paper, Section 2 introduces the new microfabrication flow and its experimental validation. Section 3 presents the polymeric piezoelectric MEMS mass sensors fabricated by the new microfabrication flow. Section 4 concludes the whole paper.

## 2. A Simple Microfabrication Flow for Polymeric Piezoelectric MEMS Devices

### 2.1. Depiction of the Processing Flow

The core idea behind the new fabrication flow is to reduce the process complexity and the turnaround time by minimizing the usage of the traditional methodology of microfabrication. Traditional micromachining processes repeat or iterate a 3-step cycle. Materials are indirectly processed layer-by-layer. The first step is material deposition, followed by masking lithography. The last step is the selective etching of the deposited material. This classic methodology is highly accurate yet complex. However, it is the only robust way to process silicon materials at a large scale, building up the basis for microfabrication flows of silicon MEMS devices and integrated circuits. For polymeric materials, the situation is different. Polymeric microstructures can be fabricated using direct patterning techniques besides the layer-by-layer micromachining flows based on the 3-step cycle. These direct processing technologies do not need masking lithography or selective etching, significantly reducing the overall process complexity. Typical examples of direct processing technologies of polymers are laser micromachining and 3D printing.

**Figure 1:**
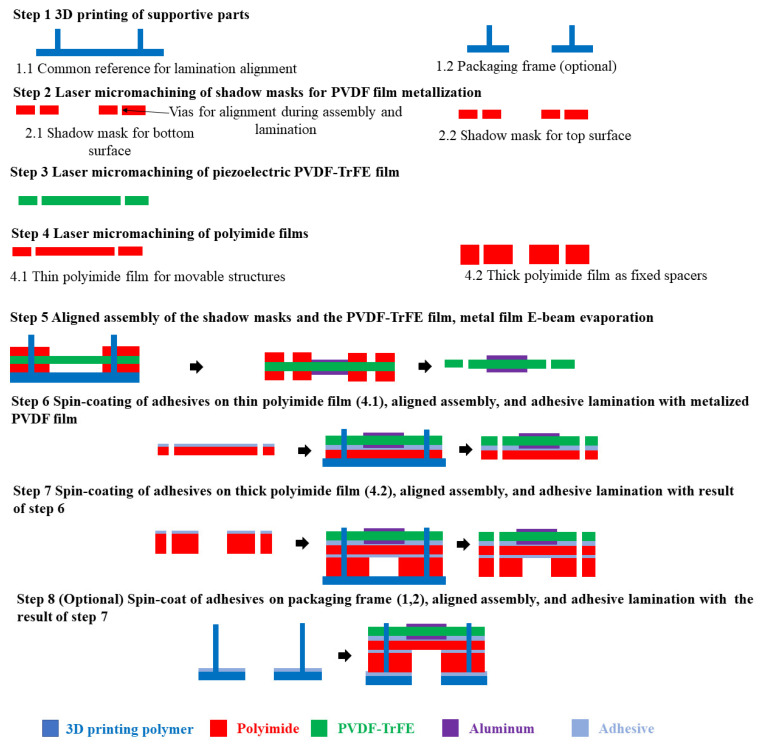
A microfabrication flow of polymeric piezoelectric MEMS devices based on laser micromachining, 3D printing, and adhesive lamination.

The microfabrication flow developed in this paper uses laser micromachining of off-the-shelf thin films for polymeric piezoelectric MEMS devices. In this new microfabrication flow, the micromechanical structure layer comprises polyimide (PI) thin films. Off-the-shelf piezoelectric polyvinylidene fluoride-trifluoroethylene (PVDF-TrFE) thin films are used as the electromechanical coupling layers. PVDF-TrFE is selected because it has a higher remanent polarization (higher piezoelectric coefficient) and higher temperature stability than PVDF [18]. Nevertheless, it is necessary to clarify here that the authors’ research interest is the new polymeric microfabrication technology and its application in MEMS devices. Every type of piezoelectric polymer thin film complaint with the direct processing technologies in this paper can be used, including PVDF. 3D printing is used to manufacture supporting structures. The microfabrication flow is shown in Figure 1.

In Figure 1, the 3D printed supporting structures are mainly used to align different thin-film layers during the assembly in the following processing steps. An alignment procedure is the basis of a robust microfabrication flow for MEMS devices. For a microfabrication flow based on the classic 3-step cycle, the accurate successive overlays of micromachined layers are implemented by using alignment markers during the masking lithography process. For the newly-developed microfabrication flow, a 3D-printed frame is used as the common reference. Each film layer has vias, cut by laser micromachining process, at the same location with reference to the position of the pillars on the alignment frame. Consequently, different films can be overlaid accurately during the assembly process.

The fabrication flow in Figure 1 uses poled PVDF-TrFE thin films for piezoelectric electromechanical coupling. It is challenging to conduct metallization processes using surface micromachining, such as the lift-off, on thin films without damaging them. Hence, metallization based on shadow masks is used for the electrode layers.

Polymeric piezoelectric MEMS transducers made by the new microfabrication flow in Figure 1 do not have substrates underneath the movable microstructures. The bottom surface of the device is fully exposed. It can be further functionalized to fit specific practical applications.

The fixed constraint condition of the MEMS devices fabricated by the technology in Figure 1 relies mainly on the thick polyimide spacer layer. The fabricated MEMS devices can be considered flexible MEMS devices. If an application scenario needs supplementary constraints, extra frames can be 3D printed, as shown in Step 9 in Figure 1.

### 2.2. MEMS Design to Test Fabrication Technology Robustness

**Figure 2:**
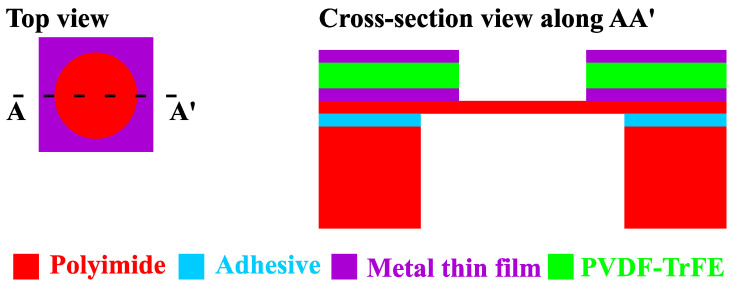
Schematic of the MEMS test structures for the newly-developed microfabrication technology.

MEMS devices based on circular membranes are selected as the test structures to validate the robustness of the new microfabrication flows. This type of microstructure is frequently used for the atmospheric two-step resonating MEMS mass sensors [3,4,7]. The schematic of the test structures is shown in Figure 2.

**Figure 3:**
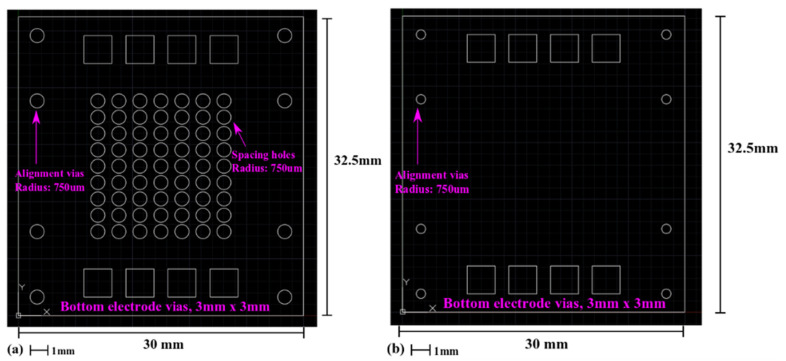

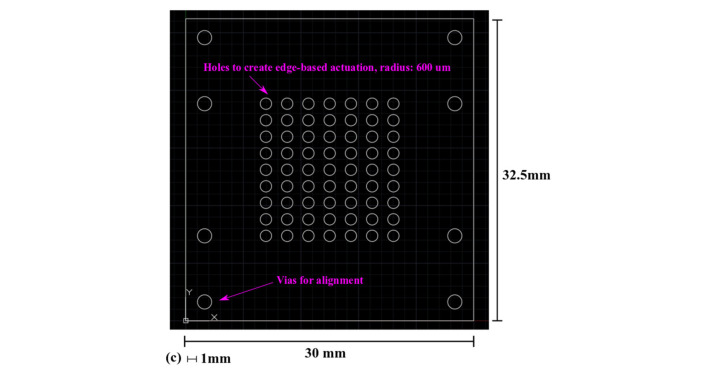
Mask designs for the laser micromachining processes of the polymeric piezoelectric MEMS devices for robustness test. (**a**) Polyimide spacer layer; (**b**) Polyimide structure layer; and (**c**) PVDF layer.

In Figure 2, the PVDF-TrFE layer is designed to partially cover the membrane area, implementing an edge-based electromechanical coupling mechanism. Edge-based actuation is more effective for the fundamental vibration mode used for mass sensing. The effective mass of membrane MEMS resonators is concentrated around the center of the membrane. This region has more significant amplitude dynamics. Partial, edge-only coverage of the PVDF-TrFE membrane can reduce the effective mass for the fundamental vibration mode, increasing the sensor’s resonant frequency and sensitivity to mass perturbations. The mask designs for the laser micromachining processes of the polyimide layers and the PVDF-TrFE layer are shown in Figure 3.

The MEMS device in Figure 3 is designed as an array of 63 circular MEMS transducers. The standard deviation in the resonant frequency of these transducers will provide information regarding the reproducibility of the microfabrication process described in Figure 1. During a laser micromachining process, the laser dot will move along the white lines in Figure 3, removing the material within a closed shape. The spacer design in Figure 3a will create holes in the spacer layer with a radius of 750 µm. After the spacer layer in Figure 3a is combined with the structural layer in Figure 3b, movable micro membranes without substrate underneath can be created. The test MEMS structures are designed to resonate at their fundamental mode.

In Figure 3c, circular holes (radius: 600 µm) are created on the PVDF-TrFE layer. They are concentric with the holes in Figure 3a to implement a 150 µm-wide piezoelectric actuation ring.

### 2.3. Fabrication of the MEMS Test Structures

The 3D printing of the alignment frames is done at 3Dshops^@^, a local 3D printing service provider, using stereolithography 3D printers and materials from Formalabs^@^. All other components are fabricated using equipment in the authors’ research facility. The information on materials used to fabricate the MEMS test structures is listed in Table 2.

The off-the-shelf PVDF-TrFE film is poled by the manufacturer. It is already piezoelectric when it arrives at the authors’ laboratory. Material properties in Table 2 are also available on PolyK’s (the manufacturer) website. Similarly, the mechanical properties of the polyimide films are based on the data from Dupont’s website. Polypropylene carbonate (PPC) in Table 2 is used as adhesive. This polymer has been widely used as a biocompatible and environmentally-friendly binding agent [19,20,21]. The specific equipment used to fabricate the test MEMS structures and the main process parameters are summarized in Table 3.

### 2.4. Characterization of Fabricated MEMS Test Structures for Technology Validation

#### 2.4.1. Optical Inspection

**Figure 4:**
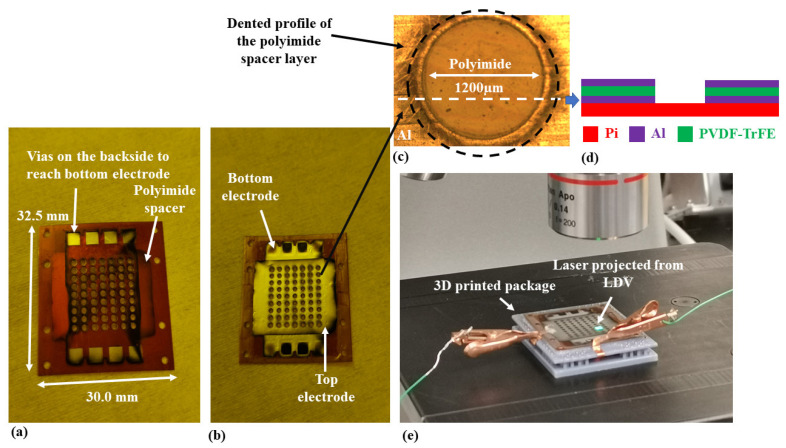
Optical images of fabricated MEMS test structures to evaluate the technology’s robustness. (**a**) The backside of the device, (**b**) The front side of the device, (**c**) Zoomed image of a single transducer, (**d**) Illustrated cross-section views of layers along the dashed line, and (**e**) Packaged MEMS transducer array during the measurement of electrically-driven mechanical resonance.

After the fabrication, the MEMS test structures were first optically inspected to detect possible fabrication-related issues. The corresponding images are shown in Figure 4.

As shown in Figure 4a,b, the bottom and top electrode layers are deposited orthogonally to each other. The interface of the bottom electrode layer is on the backside of the device. The interface of the top electrode layer is on the front. Such a configuration avoids short circuits during the packaging phase.

In Figure 4b, though the layout of the MEMS transducers to evaluate the newly-developed microfabrication lows are designed as an array, the bottom and top electrode layers connect all membranes in parallel. When an electrical signal is provided, these membrane MEMS transducers will be actuated simultaneously, with the whole array resonating as a single device.

As shown in Figure 4c, the lamination process has created visible traces of the polyimide spacer profile on the metalized PVDF-TrFE layer. The alignment accuracy during the microfabrication process can be evaluated by comparing the position of the traces and the profile of the holes on the PVDF-TrFE layer. The two circles are almost concentric, indicating that the polymeric films made by masks in Figure 3 have been aligned accurately. This result supports the effectiveness of the alignment method using a 3D printed reference frame. Figure 4d illustrates the cross-section view of the films on the top surface of the structural layer.

In Figure 4e, the 3D printed package does not provide extra fixed constraints. It is only used as a frame to hold the MEMS test structures for further characterizations.

#### 2.4.2. Mechanical Resonance Measurement

**Figure 5:**
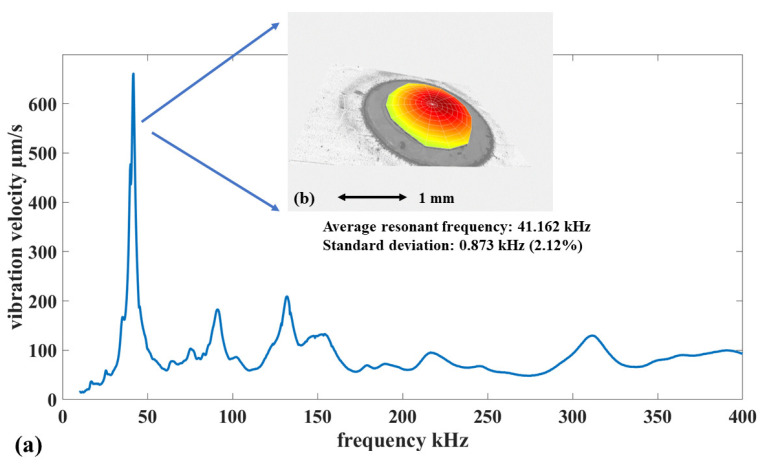
The mechanical resonance measurement result of the MEMS test structures for the microfabrication technology. (**a**) The average spectrum of mechanical resonance for 63 circular membrane MEMS transducers. (**b**) The representative fundamental resonance mode.

After the optical inspection, the mechanical resonant frequency of all MEMS transducers within the array was measured by a Polytec^@^ MSA-500 Laser Doppler Vibrometer (LDV). An LDV measurement can provide high-resolution, accurate, and comprehensive information about the mechanical resonance, such as amplitude, frequency, and modal shape. During the LDV measurement, the MEMS test structures were actuated by an AC periodic chirp signal with a peak-to-peak amplitude of 3 V. The average spectrum of all 63 test MEMS transducers is shown in Figure 5.

In Figure 5a, the most significant resonance peak is identified as the fundamental mode because of its modal shape. The fundamental resonant mode of a fully-clamped circular membrane has a Gaussian shape. Figure 5b shows the measured modal shape for the most dominant mechanical resonance response. It has a typical Gaussian shape. This result means that the effective boundary conditions of the test structures correspond to fully-clamped circular membranes.

Mechanical resonance peaks for higher modes are also visible in Figure 5a. Their magnitude is significantly lower than the peak for the fundamental mode. Their appearance is a result of edge-based actuation. The LDV measurement result by far demonstrates that the adhesive lamination of the new fabrication technology has not created significant structural defects. The mechanical resonance of the test structures matches well with the design specification.

In Figure 5a, the average resonant frequency of the 63 MEMS transducers is 41.162 kHz, with a standard deviation of 2.12%. The statistical variability of the measurements over the test structures indicates that the critical steps of the new microfabrication flow can lead to reproducible and reliable devices. This conclusion is based on a comparison with the authors’ previous research. In the past, the authors developed polymeric MEMS fabrication technologies based on the surface micromachining of the SU-8 photoresist. These microfabrication flows use the classic methodology based on the 3-step cycle, i.e., material deposition, masking lithography, and selective etching. The reproducibility of these fabrication flows has been extensively validated with different MEMS structures, with a corresponding standard deviation between 1.5% to 3% [22,23,24,25]. The 63 MEMS transducers fabricated by the newly-developed fabrication technology in this paper have their resonant frequency standard deviation close to the lower limit of this range. This result indicates that the reproducibility of the newly-developed microfabrication flow is at the same level as the layer-by-layer surface micromachining techniques for polymeric MEMS devices.

Overall, the experimental characterization of the MEMS test structures has validated the robustness of the microfabrication flow in Figure 1. It can be used to manufacture MEMS devices dedicated to specific applications.

## 3. Piezoelectric MEMS Mass Sensors Based on Polymeric Thin Films

### 3.1. Designs for the Piezoelectric Mass Sensor

**Figure 6:**
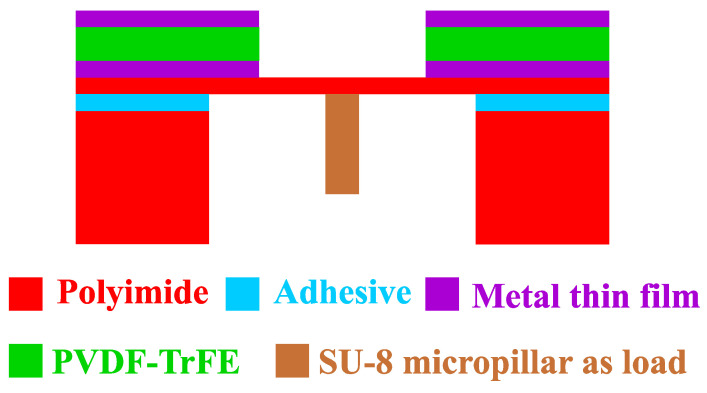
Cross-section view of the MEMS mass sensor to be fabricated.

The cross-section schematic of the piezoelectric mass sensor and its testing method is shown in Figure 6.

As shown in Figure 6, the polymeric piezoelectric MEMS mass sensor uses a similar structural design as the MEMS test structures in Figure 2. The SU-8 micropillar is used as a test load for the preliminary test of the performance of the mass sensors. The SU-8 micropillar will be made by a lithography process on the bottom polyimide surface of the movable membrane. This lithography will be carried out before the adhesive lamination that forms the final devices.

To avoid confusion, it is necessary to clarify that the primary goal of loading the SU-8 rigid mass samples is to test the sensitivity of the mass sensors for a proof-of-concept. The authors did not use a specific functional coating to detect particular materials from the surrounding environment. In practice, biomass would be loaded by the classic two-step method mentioned in Section 1.

**Figure 7:**
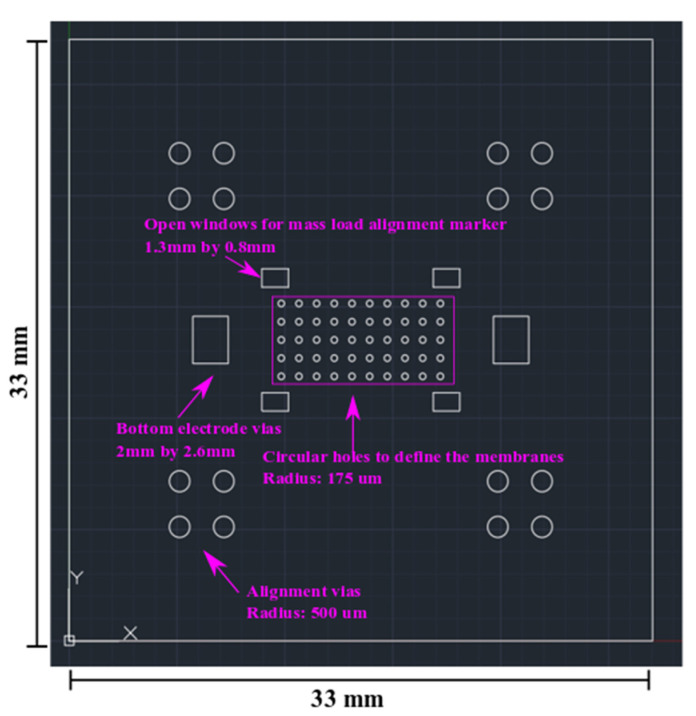
Mass sensor polyimide spacer layer laser micromachining design.

**Figure 8:**
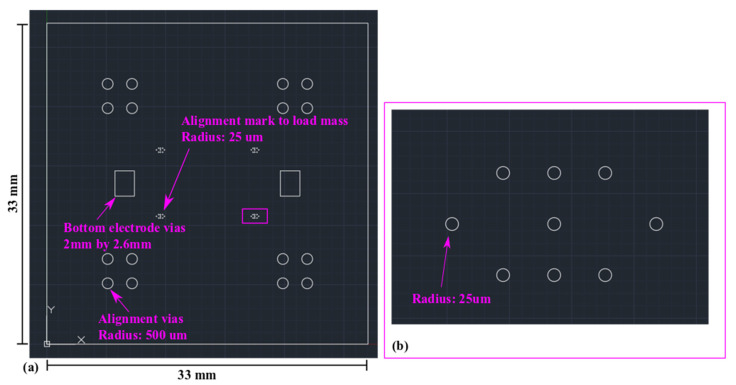
Mass sensor structural layer laser micromachining mask. (**a**) The overall view. (**b**) The zoom-in view of the mass-loading alignment markers.

**Figure 9:**
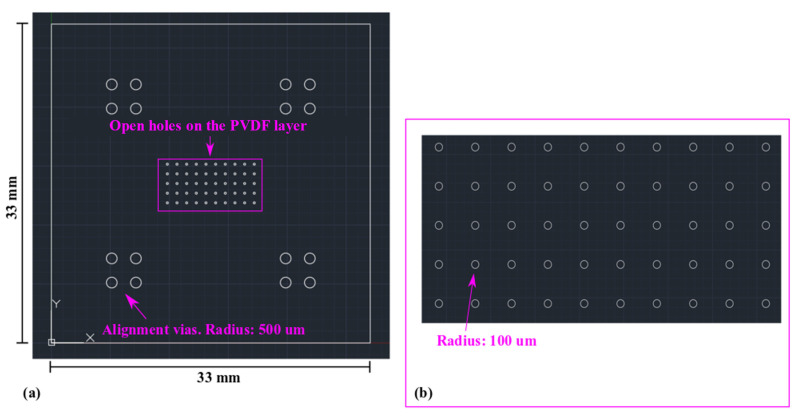
Mass sensor piezoelectric PVDF layer laser micromachining mask. (**a**) Overall view. (**b**) Zoom-in view of the circular open windows.

The laser micromachining mask designs of the mass sensors are shown in Figure 7, Figure 8 and Figure 9.

As shown in Figure 7, the piezoelectric mass sensor has an overall area of 33 mm by 33 mm. A 10-by-5 array of circular MEMS mass sensors is designed in the central region. The circular MEMS mass sensor has a radius of 175 µm. Similar to the MEMS test structures in Section 2, the mass sensors are designed to be piezoelectrically actuated and resonate at their fundamental mode. Compared with the MEMS test structures in Section 2, the dimension of the mass sensors is significantly reduced. The purpose is to increase the resonant frequency and sensitivity.

In Figure 8, four extra sets of circular vias are designed as the alignment marker for SU-8 lithography on the structural polyimide layer. Similar to the test MEMS structures in Section 2, the mass sensors also use edge-based actuation. As shown in Figure 9, the radius of the holes on the PVDF-TrFE layer is 75 µm. Correspondingly, the actuation ring is 100 µm wide.

**Figure 10:**
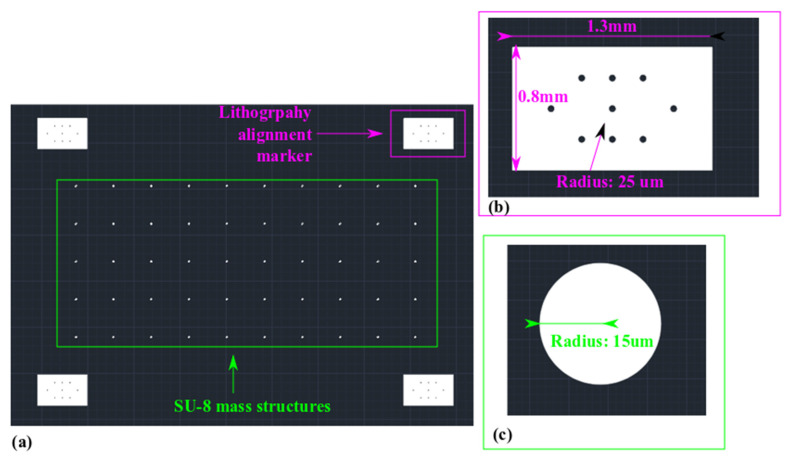
Lithography mask for SU-8 mass load. (**a**) Overall view. (**b**) Zoom-in view of the alignment markers. (**c**) Zoom-in view of a single mass load.

The lithography mask design for the SU-8 mass load is shown in Figure 10.

SU-8 photoresist is a crosslinking-based negative photoresist epoxy. The polymerization will be triggered within the exposed region. The white patterns in Figure 10 will be exposed during the SU-8 lithography process to form the circular micropillar as mass load. The radius of the micropillar is 15 µm. Its position should be around the center of the circular membrane.

### 3.2. Fabrication of the Polymeric Piezoelectric MEMS Mass Sensors

Two piezoelectric MEMS mass sensor arrays are fabricated and tested. One array is not loaded with any mass. It is used as a reference. The other one is fully loaded with SU-8 micropillars. Each mass sensor within the array will be loaded with one SU-8 micropillar.

The microfabrication flow in Figure 1 is slightly revised for the mass sensor arrays. However, the material selection and the processing recipes remain the same as in Table 2 and Table 3. For the MEMS mass sensor array loaded with SU-8 micropillars, the lithography of SU-8 2075 for the micropillars is conducted on the structural layers after Step 4 in Figure 1. The sequence of Step 6 and Step 7 in Figure 1 is reversed for both the loaded and unloaded array. The lamination between the polyimide structural and spacer layers is conducted first. Then, the metalized PVDF-TrFE layer is laminated onto the polyimide microstructures. This modification aims to protect the SU-8 microstructures during the lamination process.

The mass sensor fabrication uses the same material and recipes listed in Table 2 and Table 3. The SU-8 lithography is conducted with SU-8 2075. The corresponding recipe is summarized in Table 4.

### 3.3. Characterization of the Piezoelectric MEMS Mass Sensors

#### 3.3.1. Optical Inspection

**Figure 11:**
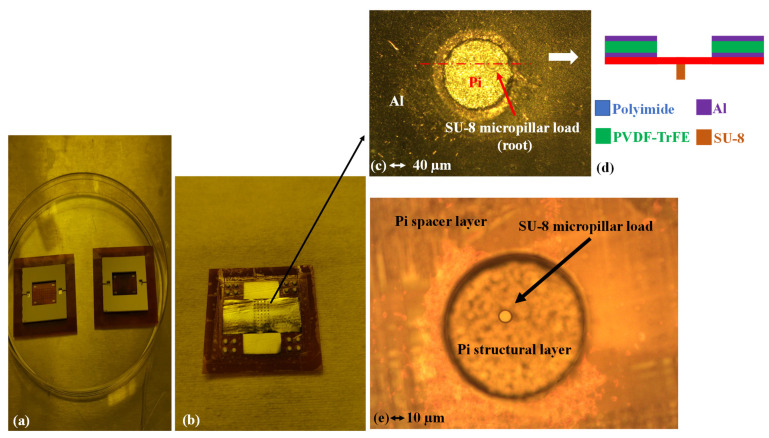
Optical images of the piezoelectric mass sensor arrays. (**a**): the two MEMS sensor arrays after the complete microfabrication flow, backside. (**b**): The front side for one of the devices before being fixed to the support frame. (**c**) Darkfield microscopic image of a single mass sensor with SU-8 micropillar load, front side. (**d**) Illustration of cross-section view along the red dashed line. (**e**) Yellow microscopic image of a single mass sensor with SU-8 micropillar load, backside.

The mass sensors are optically inspected first. The primary goal is to check the final status of the SU-8 micropillar loads. The corresponding images are shown in Figure 11.

Figure 11a shows the backside of the two polymeric piezoelectric MEMS mass sensor arrays. The white parts are 3D-printed frames to hold the sensor arrays during the measurement. They do not provide extra boundary constraints. Figure 11b shows the front side of one mass sensor array. Similar to the MEMS test structures in Section 2, the bottom and top aluminum electrode layers were deposited orthogonally to each other to minimize the risk of short-circuiting. As shown in Figure 11b, all membranes within a single array are electrically connected in parallel. An electrical signal will simultaneously actuate all of them, making the entire array operate as a single device.

Figure 11c,e shows the typical microscopic image of the front side and backside of a single MEMS membrane mass sensor. Figure 11d illustrates the cross-section along with the red dashed line in Figure 11c which serves as a graphic annotation. In Figure 11c, darkfield microscopy is used to make the SU-8 micropillar load visible from the front side. It can be observed from Figure 11c that the SU-8 micropillar is attached firmly to the bottom surface of the polyimide structural layer. No significant deformation or collapsing of the SU-8 micropillar can be found in Figure 11c. A similar result could be observed in Figure 11e. These observed results indicate that the SU-8 micropillars have survived the lamination-based assembly.

However, a misalignment between the location of the micropillar load and the geometric center of the membrane can be spotted in both Figure 11c,e. Though this misalignment is within fabrication tolerance, this location deviation might result in a higher statistical variance of the equivalent dynamic mass associated with the fundamental mode. Consequently, the standard deviation in the resonant frequency measurement might be more significant among the 50 loaded resonating MEMS mass sensors. The actual impact on the performance of the polymeric piezoelectric MEMS mass sensor array can only be evaluated through LDV measurement.

#### 3.3.2. Mass Estimation for the SU-8 Micropillar Load and the MEMS Mass Sensor

**Figure 12:**
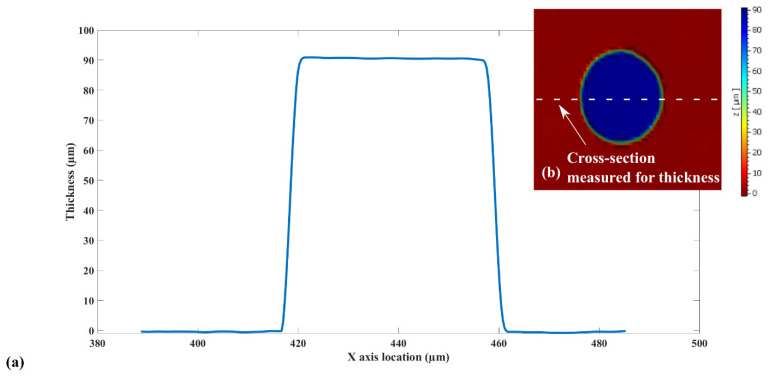
SU-8 mass load white-light interferometer measurement result. (**a**) The thickness measurement. (**b**) The SU-8 pillar measured.

After inspecting the final status of the SU-8 micropillars, the actual dimensions of these microstructures are measured. They are the basis for an accurate estimation of the loaded mass and correspondingly, the sensitivity of the mass sensors. The dimension of the SU-8 micropillars has been measured by a white light interferometer. The topological scanning module of the Polytec^@^ MSA-500 LDV is used to conduct this characterization. The representative result is shown in Figure 12.

As shown in Figure 12, the SU-8 lithography recipe in Table 4 has resulted in a structural thickness of around 90 µm. The pillar load’s actual radius is around 22 µm. SU-8 2075 is highly viscous; it mainly consists of polymer epoxy and photoinitiator salt. Hence, it is reasonable to directly use SU-8 2075′s density, 1230 kg/m^3^, to estimate the load mass. The computed mass for a single SU-8 pillar load is around 167 ng.

The rigid mass of a single piezoelectric MEMS mass sensor is estimated using the total mass of the polyimide circular area exposed through the hole on the PVDF-TrFE layer. According to Figure 9, the radius of the exposed circular area is 75 µm. According to Table 2, the thickness of the structural layer is 25 µm. The density of the Kapton^@^ polyimide film provided by Dupont^@^ is 1420 kg/m^3^. Correspondingly, the rigid mass of a single resonating MEMS mass sensor is around 631.75 ng.

#### 3.3.3. Mechanical Resonance Measurement of the MEMS Mass Sensors

**Figure 13:**
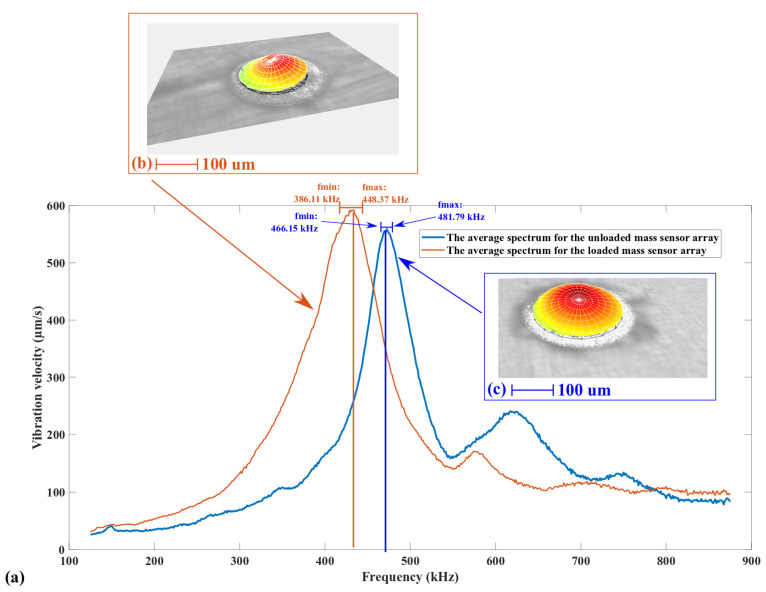
Mass sensor average performance test result. (**a**) The average spectrums for the mechanical resonance of 50 piezoelectric MEMS mass sensors, with and without the load. (**b**) The representative modal shape for the fundamental resonant mode of 50 MEMS mass sensors with mass load, and (**c**) the representative modal shape for the fundamental resonant mode of 50 MEMS mass sensors without mass load.

The two piezoelectric MEMS mass sensor arrays have their mechanical resonant frequency optically measured by the MSA-500 LDV. A periodic chirp signal with an AC peak-to-peak amplitude of 5 V is used to piezoelectrically actuate the MEMS sensors. For each array, all 50 membranes are measured for their resonant frequency. The average spectrums of the mechanical vibration response for the two mass sensor arrays (the reference array and the array with added rigid mass) are plotted in Figure 13. The statistics of the measured mechanical resonance are provided in Table 5.

In Figure 13, it can be confirmed that the reference array (resonating MEMS mass sensors without micropillar loads) resonates with a fundamental mode shape corresponding to a fully-clamped circular membrane. The modal shape in Figure 13c corresponds to the most prominent resonant peak in Figure 13a. It has a typical Gaussian shape, matching well with the fundamental modal shape of a fully-clamped circular membrane. In Table 5, the 50 unloaded MEMS mass sensors have an average resonant frequency of 473.97 kHz with a standard deviation of around 1.65%. The statistic information shows that the resonance behavior among the 50 unloaded MEMS mass sensors is uniform, demonstrating, once again, the robustness of the newly-developed microfabrication flow.

The most significant resonant mode of the mass sensor array loaded with the SU-8 micropillar load is also the fundamental mode. Figure 13b shows the modal shape of the most significant resonant peak of the loaded MEMS sensors, modified by the added mass perturbation. In Table 5, the average resonant frequency of the 50 MEMS mass sensors loaded with SU-8 micropillars was reduced to 417.24 kHz. Meanwhile, the uniformity in the resonance behavior was also reduced. The standard deviation of the resonant frequency increased to 7.46%. One possible reason for the significantly-reduced uniformity could be the variability induced by the misalignment between the SU-8 micropillar and the geometric center of the mass sensor. This misalignment has already been shown in Figure 11c,e.

In Figure 13a, the MEMS mass sensors loaded with SU-8 micropillars have a lower average resonant frequency and higher average resonant amplitude for the fundamental mode. This measurement result can be explained by the excitation conditions of the MEMS devices and some basics related to forced mechanical vibrations.

During the measurement of mechanical resonant behaviors of the MEMS mass sensors, the electrical signals from the LDV drive the MEMS sensors into forced vibration status. These electrical voltage signals have different frequency values but the same magnitude. As a result, the mechanical actuation forces generated by the inverse piezoelectric effect have the same magnitude, but their frequency varies.

A MEMS mass sensor can be considered a second-order m-b-k system. The vibration amplitude of the MEMS mass sensors will reach its maximum value when the frequency of the actuation force is the same as its fundamental resonant frequency. Its fundamental resonant frequency and the resonant magnitude at the resonant frequency during a forced vibration can be expressed as:

(1) $$\left\{\begin{array}{l} \omega_{0}=\sqrt{\frac{k_{eq}}{m_{eq}}}\\ R(\omega_{0})=\frac{A}{b\omega_{0}} \end{array}\right.$$

In Equation (1), *ω*_0_ is the fundamental resonant frequency; *k_eq_* is the equivalent spring constant; *m_eq_* is the equivalent mass; *R* is the mechanical vibration magnitude as a function of the frequency of the input; *A* is the magnitude of the mechanical actuation force; and *b* is the velocity damping coefficient.

Based on the characterization result of SU-8 micropillars in Section 3.3.2, these structures will increase the equivalent mass of the MEMS mass sensors. Meanwhile, their radius is three times smaller than the radius of the MEMS membranes. The increment on the equivalent spring constant related to the SU-8 micropillars can be expected to be more limited. According to Equation (1), the fundamental resonant frequency of the MEMS mass sensors loaded with the SU-8 micropillars can be expected to decrease. The measurement results in Figure 13a match this analysis.

The unloaded and the loaded MEMS mass sensor arrays were actuated in the atmosphere. As shown in Figure 11, there is no substrate below the bottom surface of the MEMS membranes. Hence, the damping coefficient can be considered the same for the unloaded and loaded MEMS mass sensor arrays. Meanwhile, as already discussed above, the actuation forces induced by the electrical signals from the LDV system have the same magnitude. According to Equation (1), the loaded MEMS mass sensors are expected to have a higher response due to a reduced fundamental resonant frequency. The corresponding result can be observed in Figure 13a.

In Figure 13a, the maximum resonant frequency of the 50 MEMS mass sensors loaded with SU-8 micropillars is around 448.37 kHz. The minimum resonant frequency of the 50 MEMS mass sensors without SU-8 micropillars is around 466.15 kHz. The resonant frequency distribution of the two arrays does not overlap; there is a 17.89-kHz gap in between. The data in Table 5 are still solid for evaluating the sensitivity of the MEMS mass sensors.

#### 3.3.4. Sensitivity Estimation and Comparison for the MEMS Mass Sensors

The average mass sensing sensitivity is experimentally computed in a first-order approximation, using the data provided in Table 5 regarding the measured resonant frequencies and the mass estimation in Section 3.3.2. The experimentally achieved sensitivity is around 340 Hz/ng.

For a resonating MEMS mass sensor, when the loaded mass is significantly smaller than its rigid mass, the relationship between the frequency shift and the loaded mass can be linearized to:

(2) $$\Delta f\approx -\frac{1}{2}f_{0}\frac{\Delta m}{m_{0}}$$

In Equation (2), Δ*f* is the shift *magnitude* in the mechanical resonant frequency; *f_0_* is the initial resonant frequency without any mass load; Δ*m* is the variation in equivalent mass; and *m_0_* is the original equivalent mass. Based on the statistics about resonant frequency in Table 5 and the mass estimation in Section 3.3.2, the theoretical sensitivity of the fabricated MEMS mass sensor is around 375.12 Hz/ng.

The difference between the sensitivity extracted from measurement (340 kHz/ng) and the estimated theoretical sensitivity (375.12 kHz/ng) is around 10%. Two possible reasons for the difference are given here. First, the contribution to the effective dynamical mass of the pillars depends on their position on the vibrating membrane, as they are not perfectly added in the center. The theoretical sensitivity estimation is rather a maximum sensitivity bound, assuming that the mass perturbation has a maximum effect on the resonant frequency shift. Secondly, the perturbation mass of the SU-8 micropillar is relatively large, affecting the linearization assumption used when estimating the theoretical sensitivity. An evaluation of the performance of the fabricated polymeric piezoelectric resonating MEMS mass sensor in comparison with other reported MEMS mass sensors is shown in Table 6.

In Table 6, the performance of the authors’ polymeric piezoelectric resonating MEMS mass sensor is competitive with its counterparts made by silicon micromachining technologies. Such a result indicates that the microfabrication flow developed in this paper can manufacture resonating MEMS mass sensors with acceptable performance. Meanwhile, this fabrication flow based on polymer laser micromachining, adhesive lamination, and 3D printing can bring extra advantages such as shorter turnaround time and lower fabrication costs to these polymeric piezoelectric MEMS mass sensors. This newly-developed microfabrication flow has excellent potential in manufacturing resonating MEMS mass sensors that are easily accessible or disposable.

## 4. Conclusions

This paper introduces a simple and robust method to fabricate polymeric piezoelectric MEMS mass sensors. This new method uses laser micromachining of polymeric thin films, 3D printing, and adhesive lamination to replace the traditional layer-by-layer microfabrication of polymer MEMS devices for higher process simplicity, shorter turnaround time, and lower fabrication cost. The robustness of this new microfabrication flow has been experimentally validated. MEMS devices manufactured by this technology have a standard deviation between 1.65% to 2.2% in their performance.

The microfabrication flow developed in this paper has been used for the fabrication of arrays of piezoelectrically actuated membranes. The fabricated polymeric piezoelectric MEMS mass sensors have comparable performance to their silicon counterparts made by more complex and delicate micromachining technologies.

The microfabrication technology introduced in this paper and the polymeric piezoelectric MEMS mass sensors as its products are promising in fulfilling the increasing demand for concentration sensors that are easily accessible or disposable. They can find impactful applications in many fields, such as public health, biomedical, biochemical, or environmental engineering.

For future work, functional coatings on the surface of the polymeric piezoelectric MEMS mass sensor will be implemented. The goal is to develop low-cost MEMS mass sensors with more practical applications.

## Figures and Tables

**Table 1:** Resonating MEMS mass sensors designs and corresponding applications.

MEMS Mass Sensor Design	Example of Applications	Strength	Weakness
Suspended microfluidic resonator (SMR)	Single-cell, single-DNA, and single-protein analysis [1]	Ultra-high sensitivity	Complex fabrication flow and high cost
MEMS membrane/cantilever resonators	Concentration/Distributed mass detection, such as virus, antibody, and PH value in body fluids [3,4,5,6,7]	Simple structure and low fabrication complexity	Moderate sensitivity

**Table 2:** Materials used to fabricate MEMS structures to test the quality of the new microfabrication flow.

Name	Specification					Manufacturer/Vendor
Piezoelectric PVDF-TrFE film	**Thickness**	**Piezoelectric coefficient**		**Young’s modulus**	**Density**	PolyK^@^, Philipsburg, Pennsylvania, USA
		d_33_	d_31_, d_32_			
	15 µm	>25 pC/N	8 pC/N	>2.5 GPa	1800 kg/m^3^	
Kapton HN polyimide	**Thickness**	**Poisson ratio**		**Young’s modulus**	**Density**	Dupont^@^, USA/Cole-Parmer^@^, Canada
	25 µm (membrane)	0.34		2.5 GPa	1420 kg/m^3^	
	150 µm (spacer)					
Polypropylene carbonate	Dissolved in acetone, 25 wt%					Empower Material, New Castle, DE, USA

**Table 3:** Equipment and processing parameters to fabricate MEMS test structures.

Process	Equipment	Processing Parameter					
Laser micromachining for polyimide structural layer (25 µm)	Oxford^@^ Laser system	Intensity		Repeat time		Laser moving speed	
		100%		3		0.5 mm/s	
Laser micromachining for polyimide spacer layer and polyimide shadow masks (150 µm)		100%		12		0.5 mm/s	
Laser micromachining for PVDF-TrFE (15 µm)		50%		2		2 mm/s	
E-beam evaporation of 100 nm aluminum	DeeDirector^@^ load-lock E-beam PVD system	Current			Deposition rate		
		190~210 mA			3.9 Å/s		
Spin-coating of the PPC as adhesives	Ni-Lo^@^ 5 Vaccum spin-coater	Spin-coating speed			Resulted thickness		
		2500 rpm			300~500 nm		
Adhesive laminations	Fortex Engineering^@^ Dry film laminator Model 304	Lamination speed			Lamination temperature		
		1 mm/s			80 °C		

**Table 4:** Lithography recipe to load polymeric rigid mass to test the sensitivity of mass sensors.

Equipment: Intelligent Micropatterning^@^ SF-100 Maskless Lithography System								
Spin-Coating Speed	Soft Baking		365 nm UV Exposure		Post-Exposure Baking		Developing	
1500 RPM	65 °C	95 °C	Intensity	Duration	65 °C	95 °C	Chemical	Immersion
	3 min	6 min	10 mW/cm^2^	10 s	6 min	3 min	PGMEA	10 min

**Table 5:** Statistics of the mechanical resonance measurement of the mass sensor arrays.

	50 Unloaded MEMS Mass Sensor	50 Loaded MEMS Mass Sensor
**Average resonant frequency**	473.97 kHz	417.24 kHz
**Standard deviation**	7.82 kHz (1.65%)	31.13 kHz (7.46%)

**Table 6:** Comparison of mass sensor performance.

Reference	Microstructure Type	Material	Fabrication Method	Resonant Frequency	Sensitivity
[26]	Circular membranes	Silicon	Standard micromachining flow for silicon MEMS devices	3.62 MHz	4.81 Hz/pg
This work	Circular membranes	Polyimide and PVDF	Laser micromachining and adhesive lamination	474 kHz	340 Hz/ng
[5]	Cantilever	Silicon, ZnO	Standard micromachining flow for MEMS devices	736 kHz	313 Hz/ng
[27]	Cantilever	Silicon	Standard micromachining flow for MEMS devices	44.5 kHz	40 Hz/ng
[28]	Trampoline	Silicon	Standard micromachining flow for MEMS devices	3.15 MHz	34 Hz/ng
[29]	Cantilever	Silicon, AlN	Micromachining flows based on ICP etching, and RIE	1.68 kHz	8.3 Hz/µg

## Data Availability

The data is available upon individual request.

## References

[1] De Pastina A., Villanueva L.G. (2020). Suspended micro/nano channel resonators: A review. J. Micromech. Microeng..

[2] Gabrielson T.B. (1993). Mechanical-thermal noise in micromachined acoustic and vibration sensors. IEEE Trans. Electron Devices.

[3] Hurk R.V.D., Baghelani M., Chen J., Daneshmand M., Evoy S. (2019). Al-Mo nanocomposite functionalization for membrane-based resonance detection of bovine Herpesvirus-1. Sens. Actuators A Phys..

[4] Scarpa E., Mastronardi V.M., Guido F., Algieri L., Qualtieri A., Fiammengo R., Rizzi F., De Vittorio M. (2020). Wearable piezoelectric mass sensor based on pH sensitive hydrogels for sweat pH monitoring. Sci. Rep..

[5] Joshi P., Kumar S., Jain V.K., Akhtar J., Singh J. (2019). Distributed MEMS Mass-Sensor Based on Piezoelectric Resonant Micro-Cantilevers. J. Microelectromech. Syst..

[6] Ismail A.K., Burdess J.S., Harris A.J., McNeil C.J., Hedley J., Chang S.C., Suarez G. (2006). The principle of a MEMS circular diaphragm mass sensor. J. Micromech. Microeng..

[7] Hu Z., Hedley J., Keegan N., Spoors J., Waugh W., Gallacher B., Boillot F.-X., Collet J., McNeil C. (2013). Design, fabrication and characterization of a piezoelectric MEMS diaphragm resonator mass sensor. J. Micromech. Microeng..

[8] Burg T.P., Manalis S.R. (2003). Suspended microchannel resonators for biomolecular detection. Appl. Phys. Lett..

[9] Stockslager M.A., Olcum S., Knudsen S.M., Kimmerling R.J., Cermak N., Payer K.R., Agache V., Manalis S.R. (2019). Rapid and high-precision sizing of single particles using parallel suspended microchannel resonator arrays and deconvolution. Rev. Sci. Instrum..

[10] Belardinelli P., Souza S.N.F.D., Verlinden E., Wei J., Staufer U., Alijani F., Ghatkesar M.K. (2020). Second flexural and torsional modes of vibration in suspended microfluidic resonator for liquid density measurements. J. Micromech. Microeng..

[11] Janshoff A., Galla H.-J., Steinem C. (2000). Piezoelectric mass-sensing devices as biosensors—An alternative to optical biosensors?. Angew. Chem. Int. Ed..

[12] Sone H., Okano H., Hosaka S. (2004). Picogram mass sensor using piezoresistive cantilever for biosensor. Jpn. J. Appl. Phys..

[13] Turner K., Zhang W. Design and analysis of a dynamic MEM chemical sensor. Proceedings of the 2001 American Control Conference (Cat. No. 01CH37148).

[14] Chun K.Y., Chun J. (2018). Analysis of Serum Vedolizumab Concentrations in Over 800 Patient Samples: Distribution of Drug and Anti-Drug Ab Levels and Serial Measurement Trends. Am. J. Gastroenterol..

[15] Chun K., Yang J. (2019). P108 Serum Vedolizumab and Anti-Vedolizumab Antibody: Analysis of 6500 Patient Results Using Lab Developed Electrochemiluminescent Immunoassays (ECLIA). Am. J. Gastroenterol..

[16] Lee C.C., Southgate R., Jiao C., Gersz E., Owen J.R., Kates S.L., Beck C.A., Xie C., Daiss J.L., Post V. (2020). Deriving a dose and regimen for anti-glucosaminidase antibody passive-immunization for patients with Staphylococcus aureus osteomyelitis. Eur. Cells Mater..

[17] Leeman M., Choi J., Hansson S., Storm M.U., Nilsson L. (2018). Proteins and antibodies in serum, plasma, and whole blood—size characterization using asymmetrical flow field-flow fractionation (AF4). Anal. Bioanal. Chem..

[18] Ducrot P.-H., Dufour I., Ayela C. (2016). Optimization Of PVDF-TrFE Processing Conditions For The Fabrication Of Organic MEMS Resonators. Sci. Rep..

[19] Devi M.I., Nallamuthu N., Rajini N., Kumar T.S.M., Siengchin S., Rajulu A.V., Ayrilmis N. (2019). Biodegradable poly(propylene) carbonate using in-situ generated CuNPs coated Tamarindus indica filler for biomedical applications. Mater. Today Commun..

[20] Kumar T.S.M., Rajini N., Siengchin S., Rajulu A.V., Ayrilmis N. (2019). Influence of Musa acuminate bio-filler on the thermal, mechanical and visco-elastic behavior of poly (propylene) carbonate biocomposites. Int. J. Polym. Anal. Charact..

[21] Wang D., Yu J., Zhang J., He J., Zhang J. (2013). Transparent bionanocomposites with improved properties from poly(propylene carbonate) (PPC) and cellulose nanowhiskers (CNWs). Compos. Sci. Technol..

[22] Ge C., Cretu E. (2017). MEMS transducers low-cost fabrication using SU-8 in a sacrificial layer-free process. J. Micromech. Microeng..

[23] Ge C., Cretu E. (2018). Design and fabrication of SU-8 CMUT arrays through grayscale lithography. Sens. Actuators A Phys..

[24] Ge C., Cretu E. (2019). A sacrificial-layer-free fabrication technology for MEMS transducer on flexible substrate. Sens. Actuators A Phys..

[25] Ge C., Cretu E. (2020). A Simple and Robust Fabrication Process for SU-8 In-Plane MEMS Structures. Micromachines.

[26] Burnett R., Harris A., Ortiz P., Hedley J., Burdess J., Keegan N., Spoors J., McNeil C. (2012). Electronic detection strategies for a MEMS-based biosensor. J. Microelectromech. Syst..

[27] Ricciardi C., Fiorilli S., Bianco S., Canavese G., Castagna R., Ferrante I., Digregorio G., Marasso S.L., Napione L., Bussolino F. (2010). Development of microcantilever-based biosensor array to detect Angiopoietin-1, a marker of tumor angiogenesis. Biosens. Bioelectron..

[28] Lin A.T.-H., Yan J., Seshia A.A. (2012). Electrically addressed dual resonator sensing platform for biochemical detection. J. Microelectromech. Syst..

[29] Sökmen Ü., Stranz A., Waag A., Ababneh A., Seidel H., Schmid U., Peiner E. (2010). Evaluation of resonating Si cantilevers sputter-deposited with AlN piezoelectric thin films for mass sensing applications. J. Micromech. Microeng..

